# Supplementary material for: Simultaneous polydirectional transport of colloidal bipeds
Source: Nat Commun. 2020 Sep 16;11:4670. doi: 10.1038/s41467-020-18467-9 (PMC7495478; doi:10.1038/s41467-020-18467-9)
Supplement: Supplementary file 1 — Supplementary Information [file 41467_2020_18467_MOESM1_ESM.pdf]

Supplementary information: Simultaneous polydirectional transport of colloidal bipeds

Mahla Mirzaee-Kakhki,[1] Adrian Ernst,[1] Daniel de las Heras,[2] Maciej Urbaniak,[3] Feliks Stobiecki,[3] Jendrik Gördes,[4] Meike Reginka,[4] Arno Ehresmann,[4] and Thomas M. Fischer[1,*]

[1] *Experimentalphysik X, Physikalisches Institut, Universität Bayreuth, D-95440 Bayreuth, Germany*
[2] *Theoretische Physik II, Physikalisches Institut, Universität Bayreuth, D-95440 Bayreuth, Germany*
[3] *Institute of Molecular Physics, Polish Academy of Sciences, 60-179 Poznań, Poland.*
[4] *Institute of Physics and Center for Interdisciplinary Nanostructure Science and Technology (CINSaT), Universität Kassel, D-34132 Kassel, Germany*

(Dated: August 24, 2020)

SUPPLEMENTARY NOTE 1

We describe here the parallel polydirectional loops used to control the simultaneous motion of bipeds shown in both Fig. 2 of the main text and the Supplementary Movies. In all cases, the loops are shown in polydirectional transcription space $\mathcal{T}_{\text{poly}}$. The loops transport a set of l bipeds of lengths $b_{n1}, ..., b_{nl}$. Supplementary Fig. 1 shows the loops that control the motion of the parallel didirectional command $l = 2$ (a), the parallel tridirectional command $l = 3$ (b), the parallel pentadirectional command $l = 5$ (c), and the parallel hexadirectional command $l = 6$ (d). The parallel tetradirectional loop $l = 4$ is depicted in Supplementary Fig. 2.

In both figures we plot a top view of the equatorial plane in $\mathcal{T}_{\text{poly}}$, the cuts of the target unidirectional spheres $\mathcal{T}_{n1}, ..., \mathcal{T}_{nl}$ with the equatorial plane (colored concentric circles), the fences $\mathcal{F}_i$ with $i = 1, 2$, and the rays in $\mathcal{T}_{\text{poly}}$ that are transcribed into $\mathcal{T}_{\text{poly}}$ from the equatorial crossings of the parallel polydirectional loop $\mathcal{L}_{\text{p}}$ in control space $\mathcal{C}_{\text{p}}$. A fundamental parallel polydirectional loop $\mathcal{L}_p$ in $\mathcal{C}_p$ can wind in the clockwise (-) or counterclockwise (+) direction around the fence points within the smaller enclosed area. In Supplementary Fig. 1 and Supplementary Fig. 2 we show tables next to each equatorial plane indicating whether the loop winds clockwise (red) or counterclockwise (blue).

Up and down triangles are drawn at the intersections of the fences with the unidirectional transcription spheres. Triangles are up (down) if the scalar product of the tangent vector of the ray toward the triangle with the tangent vector of the fence is positive (negative). Taking the difference of up triangles and down triangles on a unidirectional arc inside a fundamental cone and multiplying with the winding sense of the fundamental loop gives the winding number of the particular unidirectional fundamental loop $\mathcal{L}_{ni}$ for the chosen target length b_{ni}. Hence, the winding numbers can be read directly from the equatorial plots. In addition, all winding numbers are listed in the tables.

In Supplementary Fig. 3 we show a complex heptadirectional loop. We aim the reader to decipher the associated winding numbers and trajectories.

Finally, in Supplementary Fig. 4 we show a complex undecasorting loop with a table comparing the experimental and theoretical winding numbers.

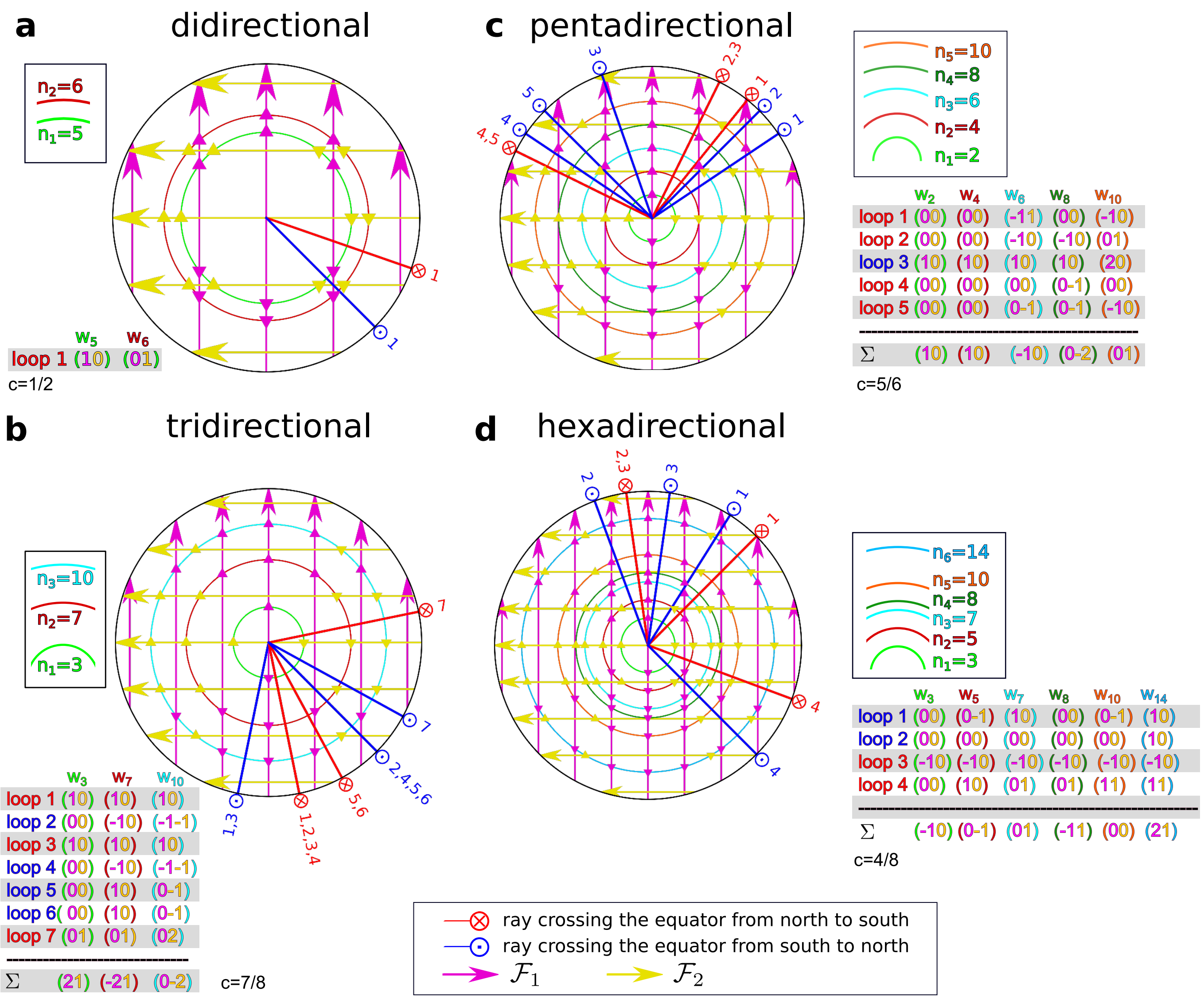

Supplementary Fig. 1. **Modulation loops in transcription space $\mathcal{T}_{\mathbf{poly}}$.** Top view of the equatorial plane of $\mathcal{T}_{\rm poly}$ showing the fences $\mathcal{F}_1$ and $\mathcal{F}_2$, the unidirectional transcription spaces (concentric circles) $\mathcal{T}_{ni}$ with $i = 1, ..., l$ and l the number of different bipeds transported by the loop, and the modulation loops of **a** the parallel didirectional command, **b** the parallel tridirectional command, **c** the parallel pentadirectional command and **d** the parallel hexadirectional command. A table indicating the winding numbers around each fence and the compaction of the loop are also depicted for each case. The procedure to read the loops is as follows. Pick the first fundamental loop 1 and determine its orientation (± 1 for mathematical positive respectively negative sense). For example, loop 1 of the parallel didirectional command (parallel hexadirectional command) has negative (positive) orientation. Next, subtract the number of pink-down triangles from that of pink-up triangles on the b_{n_1} arcs of the fundamental loop 1 and multiply it with the orientation of the loop to obtain the $\mathbf{a}_1$-displacement of the n_1-biped during the loop 1. Repeat same procedure with the yellow triangles to obtain the $\mathbf{a}_2$-displacement. Repeat this for the other lengths $b_{n_2} - b_{n_l}$ to obtain all unidirectional displacements. Count the total number of fundamental loops of the polydirectional command and compare it with the number of nearest neighbor displacements needed if multiplexing. To each polydirectional command we provide a table with the winding numbers of the fundamental loops as well as the sum of the concatenated complex polydirectional command for comparison. Fundamental loops with positive (negative) orientations are marked as blue (red) in the table.

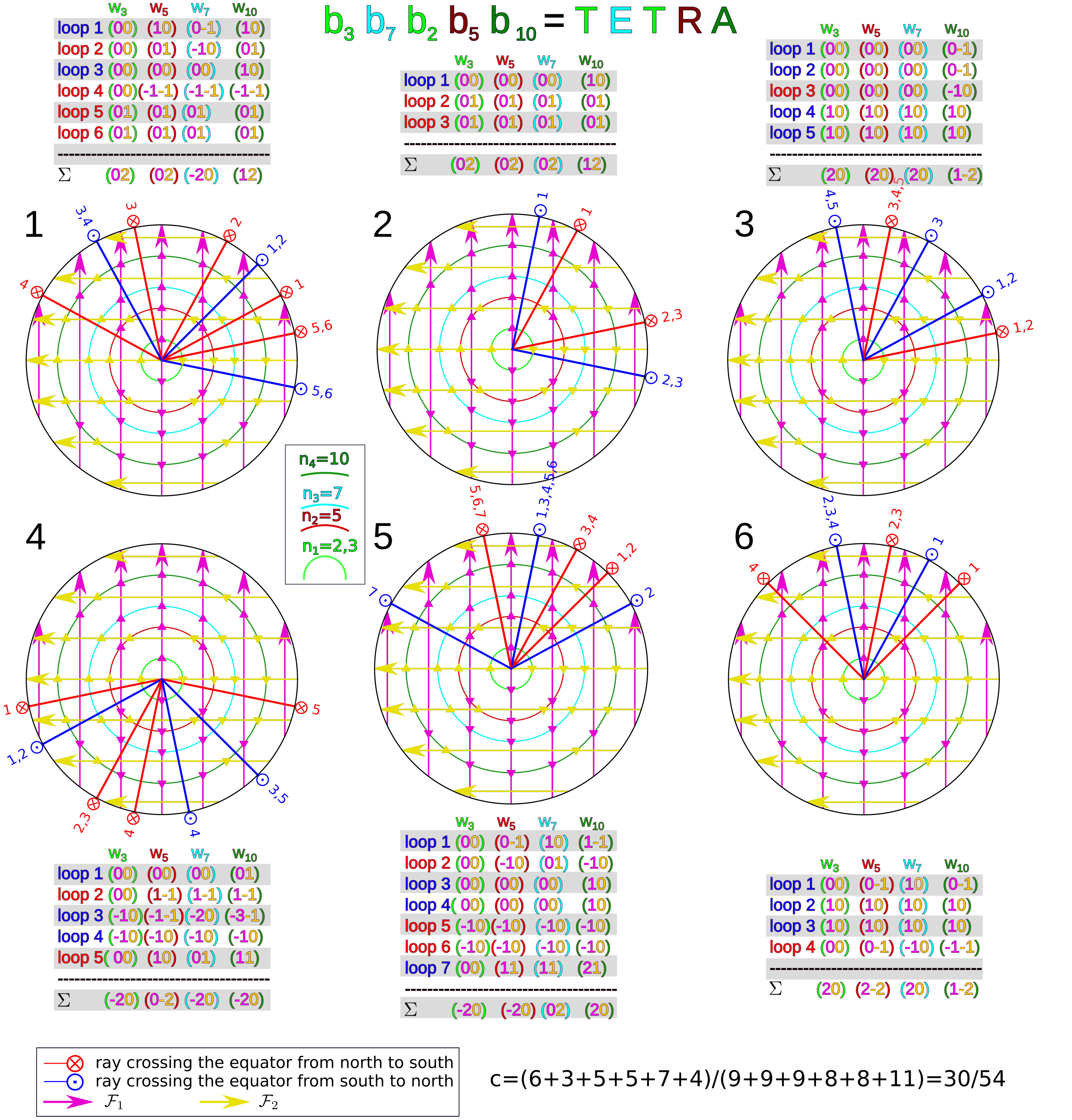

Step 1

	w_3	w_5	w_7	w_{10}
loop 1	(00)	(10)	(0-1)	(10)
loop 2	(00)	(01)	(-10)	(01)
loop 3	(00)	(00)	(00)	(10)
loop 4	(00)	(-1-1)	(-1-1)	(-1-1)
loop 5	(01)	(01)	(01)	(01)
loop 6	(01)	(01)	(01)	(01)
Σ	(02)	(02)	(-20)	(12)

Step 2

	w_3	w_5	w_7	w_{10}
loop 1	(00)	(00)	(00)	(10)
loop 2	(01)	(01)	(01)	(01)
loop 3	(01)	(01)	(01)	(01)
Σ	(02)	(02)	(02)	(12)

Step 3

	w_3	w_5	w_7	w_{10}
loop 1	(00)	(00)	(00)	(0-1)
loop 2	(00)	(00)	(00)	(0-1)
loop 3	(00)	(00)	(00)	(-10)
loop 4	(10)	(10)	(10)	(10)
loop 5	(10)	(10)	(10)	(10)
Σ	(20)	(20)	(20)	(1-2)

Step 4

	w_3	w_5	w_7	w_{10}
loop 1	(00)	(00)	(00)	(01)
loop 2	(00)	(1-1)	(1-1)	(1-1)
loop 3	(-10)	(-1-1)	(-20)	(-3-1)
loop 4	(-10)	(-10)	(-10)	(-10)
loop 5	(00)	(10)	(01)	(11)
Σ	(-20)	(0-2)	(-20)	(-20)

Step 5

	w_3	w_5	w_7	w_{10}
loop 1	(00)	(0-1)	(10)	(1-1)
loop 2	(00)	(-10)	(01)	(-10)
loop 3	(00)	(00)	(00)	(10)
loop 4	(00)	(00)	(00)	(10)
loop 5	(-10)	(-10)	(-10)	(-10)
loop 6	(-10)	(-10)	(-10)	(-10)
loop 7	(00)	(11)	(11)	(21)
Σ	(-20)	(-20)	(02)	(20)

Step 6

	w_3	w_5	w_7	w_{10}
loop 1	(00)	(0-1)	(10)	(0-1)
loop 2	(10)	(10)	(10)	(10)
loop 3	(10)	(10)	(10)	(10)
loop 4	(00)	(0-1)	(-10)	(-1-1)
Σ	(20)	(2-2)	(20)	(1-2)

Supplementary Fig. 2. **Modulation loop in transcription space $\mathcal{T}_{\mathbf{poly}}$ for the parallel tetradirectional command**. The loop induces a set of $l = 5$ bipeds to write the word TETRA as shown in Fig. 2c of the main text and the Supplementary Videos. Each letter is written in six different steps. Each equatorial plane shown in the figure represents one of these steps, as indicated. See caption of Fig. 1 for a complete explanation on how to read the fundamental loops. The word TETRA appears when the bipeds are ordered as $b_3, b_7, b_2, b_5, b_{10}$.

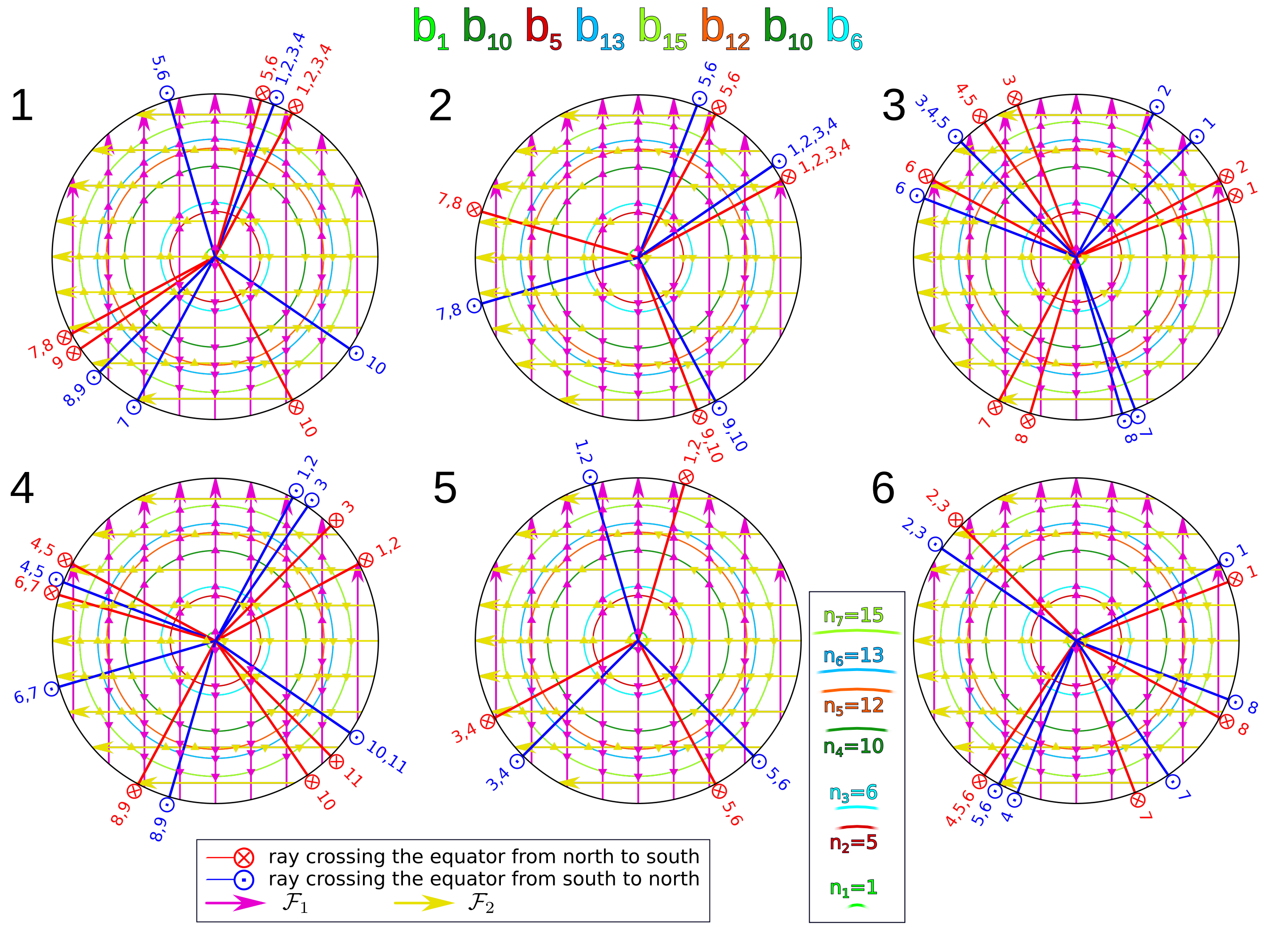

Supplementary Fig. 3. **Parallel heptadirectional command.** The loop encrypts seven letters, each one with six steps. Each equatorial plane contains the fundamental loops of one step. All fundamental loops have positive orientation here. The procedure to read the loops is the same as that explained in the caption of Fig. 1. The encrypted word appears when the bipeds are ordered as $b_1, b_{10}, b_5, b_{13}, b_{15}, b_{12}, b_{10}, b_6$. The compaction of the loop is $c = 30/54$ (see caption of Fig. 1 for an explanation on how to calculate the compaction). If you find a robust parallel heptadirectional command with less fundamental loops encoding the same word, please let us know!

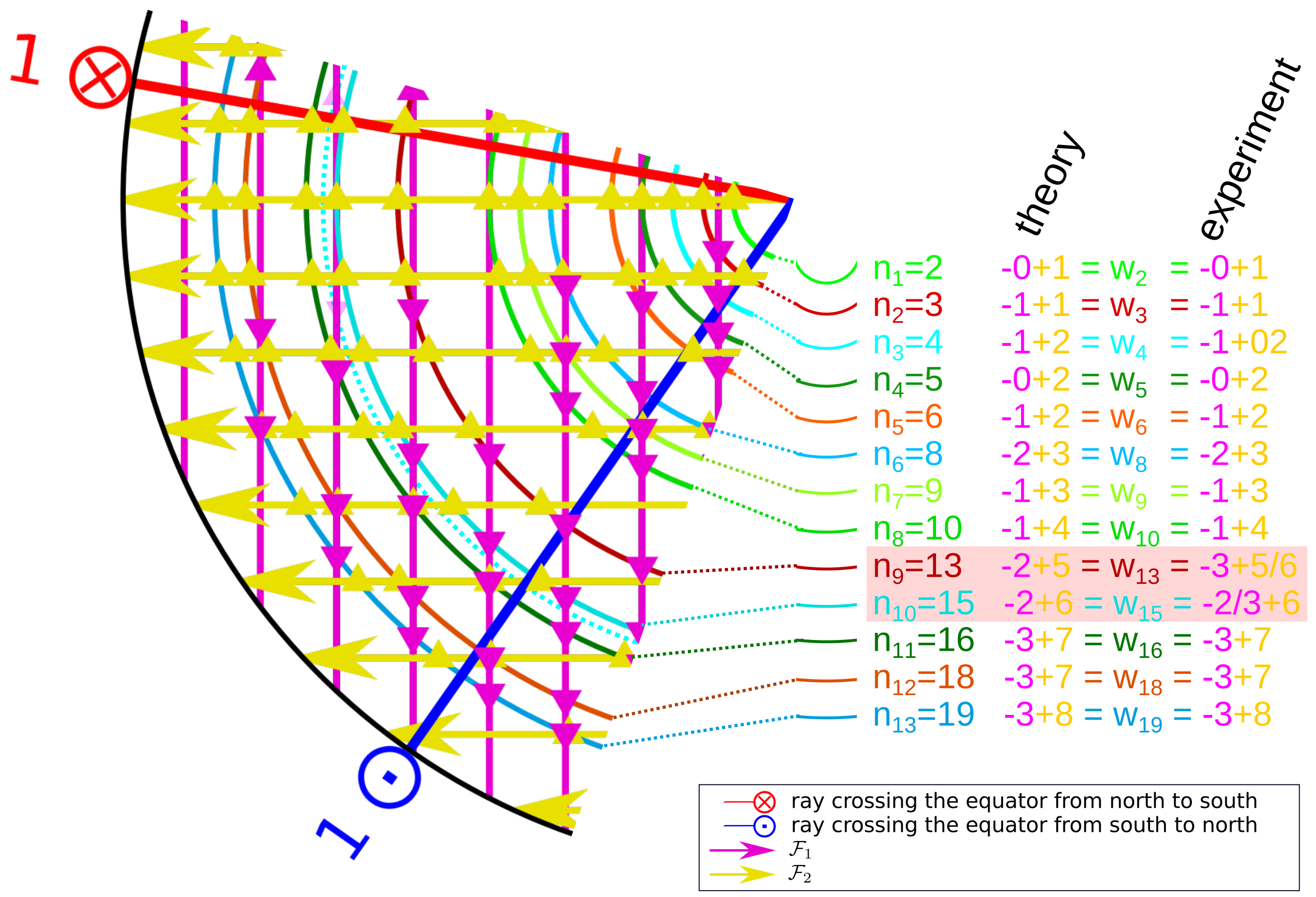

Supplementary Fig. 4. **Modulation loops in transcription space $\mathcal{T}_{\mathbf{poly}}$.** Top view of the equatorial plane of $\mathcal{T}_{\text{poly}}$ showing the fences $\mathcal{F}_1$ and $\mathcal{F}_2$, the unidirectional transcription spaces (concentric circles) $\mathcal{T}_{ni}$ with $i = 1, ..., 13$ of the thirteen different bipeds transported by the loop, and the modulation loops of parallel undecasorting command. A table indicating the theoretical winding numbers around each fence and the experimentally observed winding numbers of the motion illustrated in Fig 4 of the main text are listed for a comparison. Discrepancies in winding numbers occur for the bipeds b_{13} and b_{15}, which are marked in red in the table. An incomplete locking between $\mathbf{H}_{\text{ext}}$ and $\mathbf{b}_{13}$ due to nonadiabatic effects could be the reason for observed differences for the b_{13}-biped, for which $\mathcal{T}_{13}$, $\mathcal{F}_2$ and $\mathcal{L}_{13}$ almost fall on top of each other. The dotted transcription space is a transcription space for biped size $b_{15.3} > b_{15}$, that would contain an extra crossing of the fence $\mathcal{F}_1$, shown as transparent pink triangle demonstrating that a 2 % increase in length of the biped could explain the discrepancies between experiment an theory for the b_{15}-biped.
