# Supplementary material for: Simultaneous polydirectional transport of colloidal bipeds
Source: Nat Commun. 2020 Sep 16;11:4670. doi: 10.1038/s41467-020-18467-9 (PMC7495478; doi:10.1038/s41467-020-18467-9)
Supplement: Supplementary file 3 — Description of Additional Supplementary Files [file 41467_2020_18467_MOESM3_ESM.pdf]

Description of Additional Supplementary Files

Supplementary movie 1.mp4 Brownian dynamics simulation of a b_5 biped subject to the loop presented in figure 1 and figure 3. The foot of the biped must slide into the position that commensurate with the lattice when the control loop of the external field passes close to a fence point.

Supplementary movie 2.mp4 Reflection microscopy recording of the experiment of the didirectional command in figure 2a. To the right we show the motion of the external field in control space and the equatorial plane of the polydirectional transcription space.

Supplementary movie 3.mp4 Reflection microscopy recording of the experiment of the tridirectional command in figure 2b. To the right we show the motion of the external field in control space and the equatorial plane of the polydirectional transcription space.

Supplementary movie 4.mp4 Reflection microscopy recording of the experiment of the tetradirectional command in figure 2c. To the right we show the equatorial plane of the polydirectional transcription space. The tetradirectional command consists of six complex loops that are repeated three times and they have different rays in the equatorial planes.

Supplementary movie 5.mp4 Reflection microscopy recording of the experiment of the pentadirectional command in figure 2d. To the right we show the equatorial plane of the polydirectional transcription space.

Supplementary movie 6.mp4 Reflection microscopy recording of the experiment of the hexadirectional command in figure 2e. To the right we show the equatorial plane of the polydirectional transcription space.

Supplementary movie 7.mp4 Brownian dynamics simulation of the tetradirectional command shown in figure 2c. Corresponding experiments are shown in Supplementary Movie 4.

Supplementary movie 8.mp4 Reflection microscopy recording of the experiment of the undecasorting command in figure 4. To the right we show the equatorial plane of the polydirectional transcription space. At the end of the movie we show that the bipeds are indeed self assembled bipeds that depolymerize to monomers and dimers if we switch off the external field.

Supplementary movie 9.mp4 Visual explanation of the algorithm to find a compacted loop with the desired transport directions.
